# Supplementary material for: Dim light at night unmasks sex-specific differences in circadian and autonomic regulation of cardiovascular physiology
Source: Commun Biol. 2024 Sep 27;7:1191. doi: 10.1038/s42003-024-06861-8 (PMC11437115; doi:10.1038/s42003-024-06861-8)
Supplement: Supplementary file 2 — Supplementary Information [file 42003_2024_6861_MOESM2_ESM.pdf]

Dim light at night unmasks sex-specific differences in circadian and autonomic regulation of cardiovascular physiology

Abhilash Prabhat[1], Dema Sami[1], Allison Ehlman[1], Isabel Stumpf[1], Tanya Seward[1], Wen Su[1], Ming C. Gong[1], Elizabeth A. Schroder[1,2], Brian P. Delisle[1]

[1]Department of Physiology, University of Kentucky, Lexington, KY, USA
[2]Department of Internal Medicine, University of Kentucky, Lexington, KY, USA

Corresponding authors:

Abhilash Prabhat
(859) 323-5101
apr288@uky.edu
741 S Limestone Street BBSRB B339, Lexington, KY, 40536

Brian P. Delisle
(859) 323-2797
brian.delisle@uky.edu
741 S Limestone Street BBSRB B353, Lexington, KY, 40536

Supplementary information

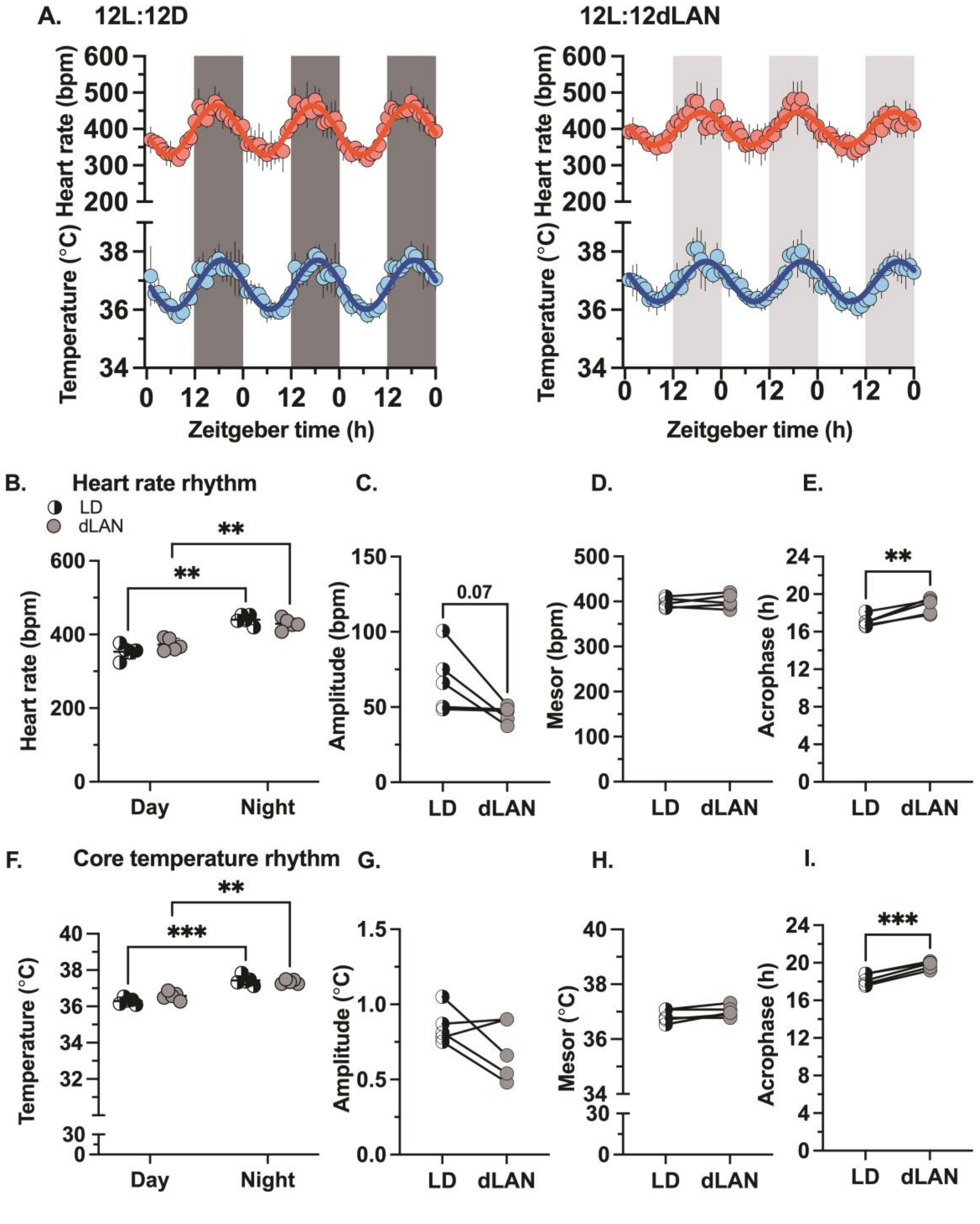

Supplementary Figure 1: dLAN effects on the heart rate and core body temperature in male mice under thermoneutrality

A. The hourly mean heart rate (HR, red) or core body temperature (Tb, blue) data measured from male mice housed in LD or dLAN plotted as a function of zeitgeber time. The mean data were fitted with a cosine function (red and blue lines). **B.** The average HR measured for each mouse housed in LD (half-filled circles) or dLAN (grey circles) during the day or night. **C-E.** The amplitude, mesor, and acrophase of the day-night rhythms in HR were calculated using cosine fit to the individual mouse data for each condition. **F.** The average Tb was measured for each mouse housed in LD (half-filled circles) or dLAN (grey circles) during the day or night. **G-L.** The amplitude, mesor, and acrophase of the day-night rhythms in Tb were calculated using cosine fit to the individual mouse data for each condition. Data are presented as a scatter plot with the mean and SD. 2-way repeated measures ANOVA followed by Sidak's post hoc test for LD and dLAN day vs. night comparisons (**B, F**). Paired t-test compared the difference between the amplitudes (**C, G**), mesor (**D, H**), and acrophase (**E, I**). $p<0.05$ is considered statistically significant. **$p<0.01$ and ***$p<0.001$.

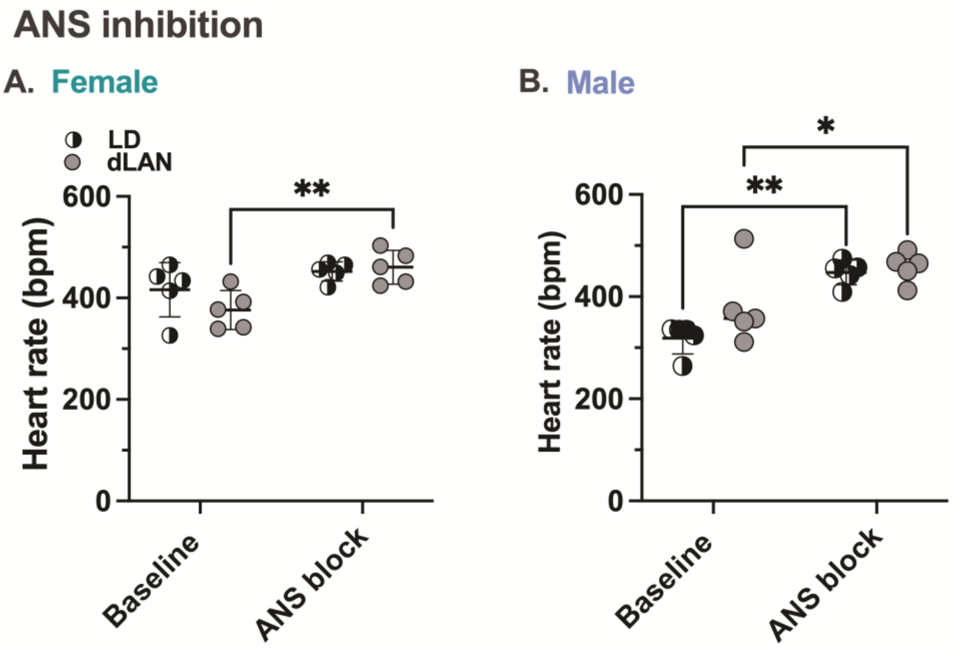

Supplementary Figure 2: dLAN did not alter the intrinsic heart rate in male and female mice after autonomic inhibition under thermoneutrality

A., B. The average mean of two hours Baseline (before) and after autonomic nervous system inhibition (ANS inhibition) heart rate for each mouse housed in LD (half-filled circles) or dLAN (grey circles) in females and males, respectively (n=5/sex). Data are presented as a scatter plot with the mean and SD. 2-way repeated measures ANOVA followed by Sidak's posthoc test for multiple comparisons. $p<0.05$ is considered as statistically significant. *$p<0.05$ and **$p<0.01$.

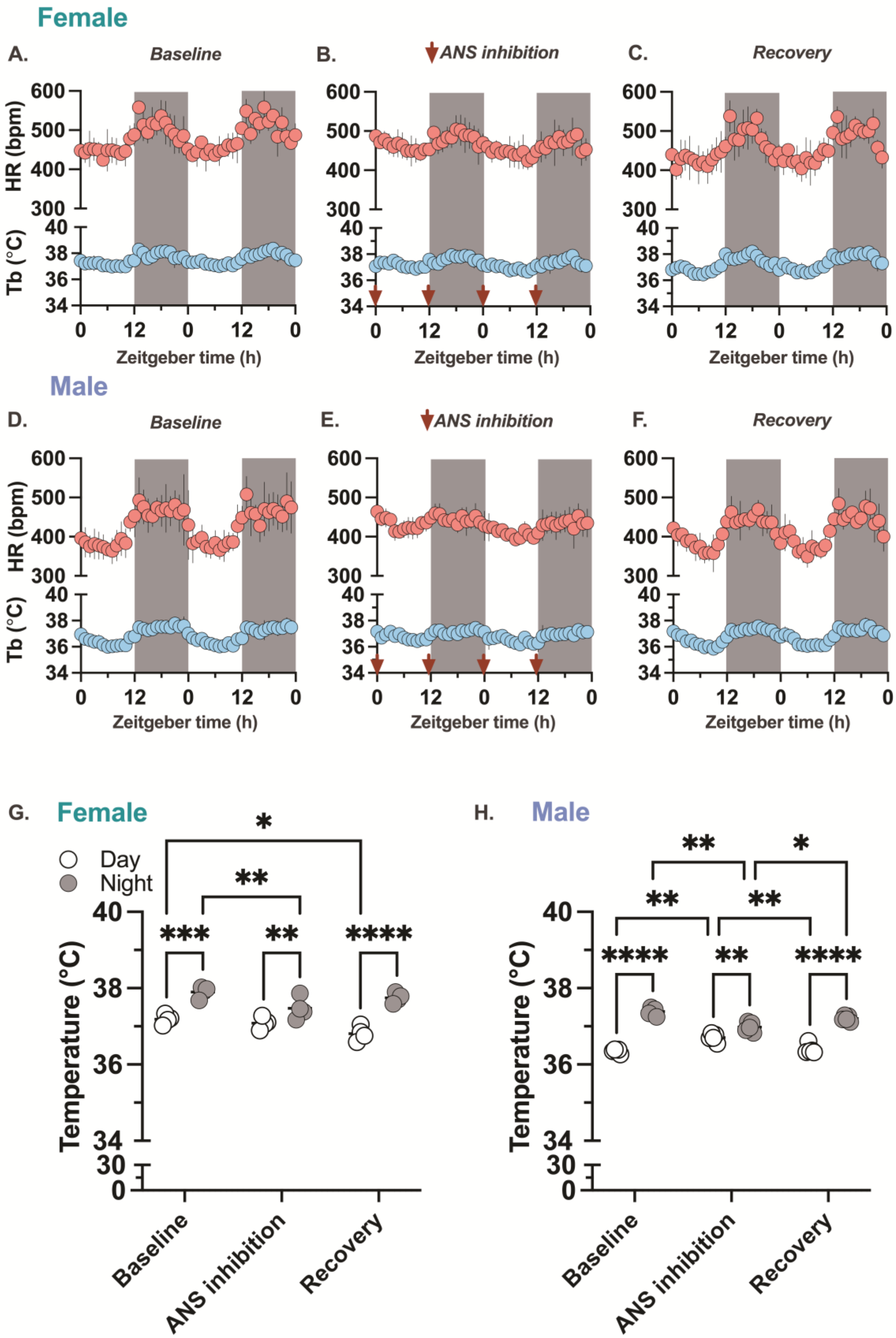
Female
A.
Baseline
B.
ANS inhibition
C.
Recovery
HR (bpm)
Tb (°C)
Zeitgeber time (h)
Male
D.
Baseline
E.
ANS inhibition
F.
Recovery
HR (bpm)
Tb (°C)
Zeitgeber time (h)
G.
Female
Day
Night
Temperature (°C)
Baseline
ANS inhibition
Recovery
H.
Male
Temperature (°C)
Baseline
ANS inhibition
Recovery

Supplementary Figure 3: Effects of autonomic inhibition on the core body temperature in female and male mice

The hourly mean for heart rate (HR, red) and core body temperature (Tb, blue) data recorded from female and male mice were plotted as a function of zeitgeber time for two consecutive days. **A., B.,** and **C.** Show the data measured before inhibition (baseline), during autonomic nervous system (ANS) inhibition, and after recovery in female mice (n=4). **D., E.,** and **F.** Show the data measured before inhibition (baseline), during ANS inhibition, and after recovery in male mice (n=5). **G., H.** The average Tb data was measured for each mouse during the day (white circle) or night (grey circle) before inhibition (baseline), during ANS inhibition, and after recovery in female and male mice. The red arrow represents autonomic nervous system inhibition (ANS inhibition) injection at zeitgeber time 23.5 and 11.5 (lights on at ZT 0). Data are presented as a scatter plot with the mean and SD. Significance was determined using a 2-way repeated measures ANOVA and Tukey's posthoc test. *$p<0.05$, **$p<0.01$, ***$p<0.001$, and ****$p<0.0001$.

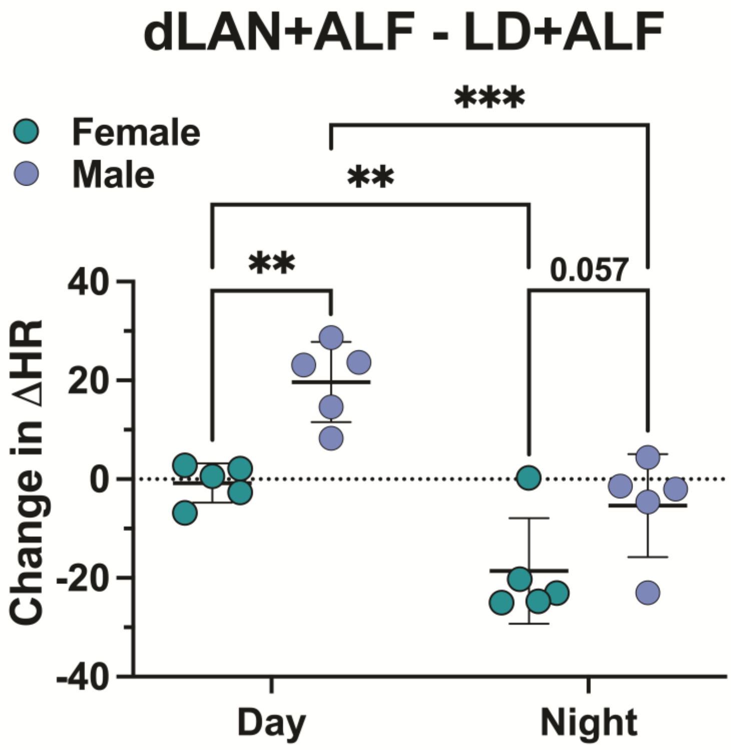

Supplementary Figure 4: dLAN causes sex differences in change in the ∆HR during the day and night.

Effects of dim light at night- The change in the ∆HR between dLAN+ALF and LD+ALF in females and males, respectively (n=5/sex). Data are presented as a scatter plot with the mean and SD. 2-way repeated measures ANOVA followed by Sidak's posthoc test for multiple comparisons. $p<0.05$ is considered as statistically significant. **$p<0.01$ and ***$p<0.001$.

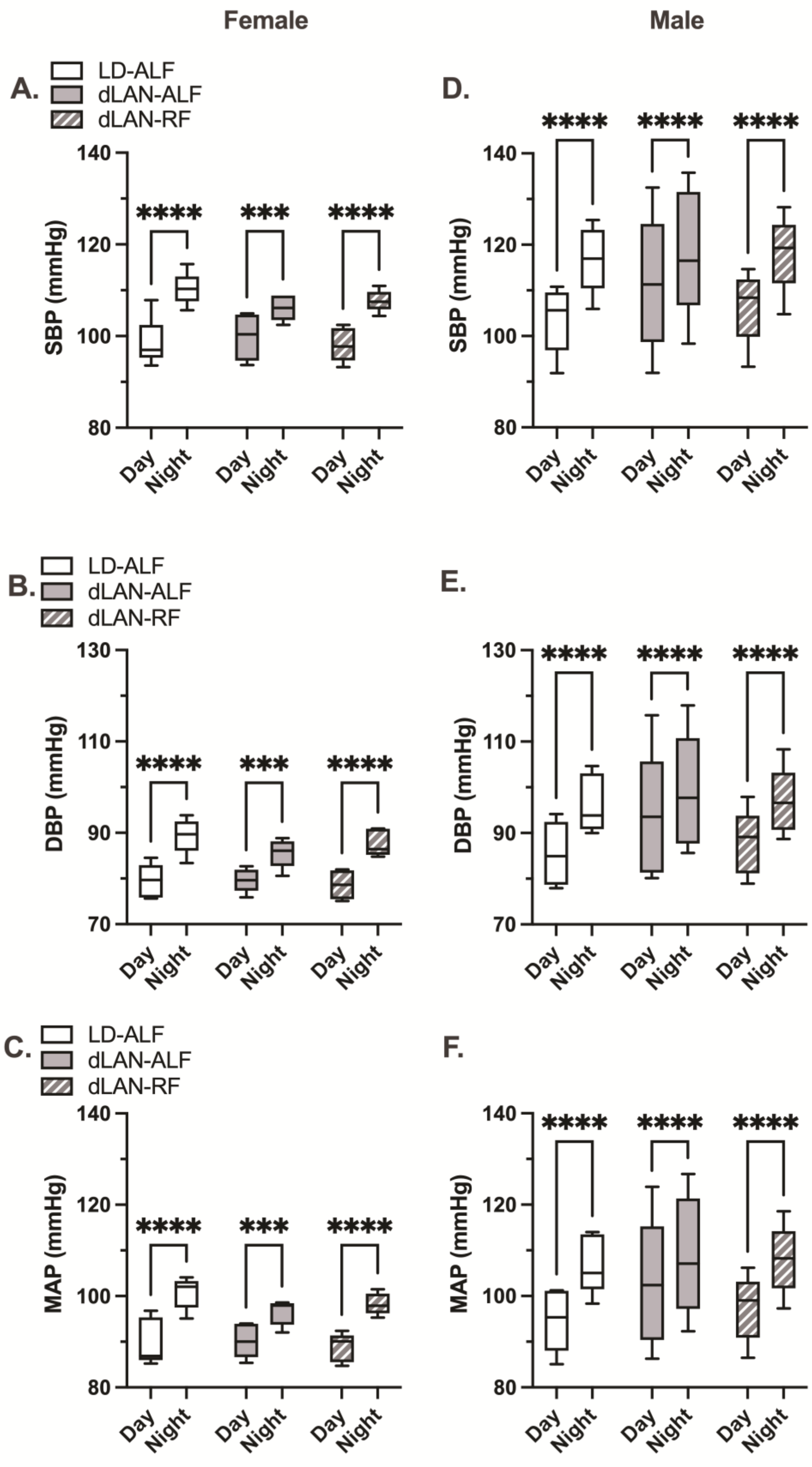

Female
Male
A.
B.
C.
D.
E.
F.
LD-ALF
dLAN-ALF
dLAN-RF
SBP (mmHg)
DBP (mmHg)
MAP (mmHg)
Day
Night
****
***

Supplementary Figure 5: dLAN effects on the day-night variation in blood pressure parameters

A., D. Show the average day and night systolic blood pressure (SBP) in female and male mice (n=5/sex) under LD+ALF, dLAN+ALF, and dLAN+RF. **B., E.** Show the average day and night diastolic blood pressure (DBP) in female and male mice. **C., F.** Show the average day and night mean arterial pressure (MAP) in female and male mice. Data are presented as box-whisker plots (median) with error bars representing minimum and maximum values. Significance was determined using a 2-way repeated measures ANOVA and Tukey's posthoc test. ***$p<0.001$ and ****$p<0.0001$.

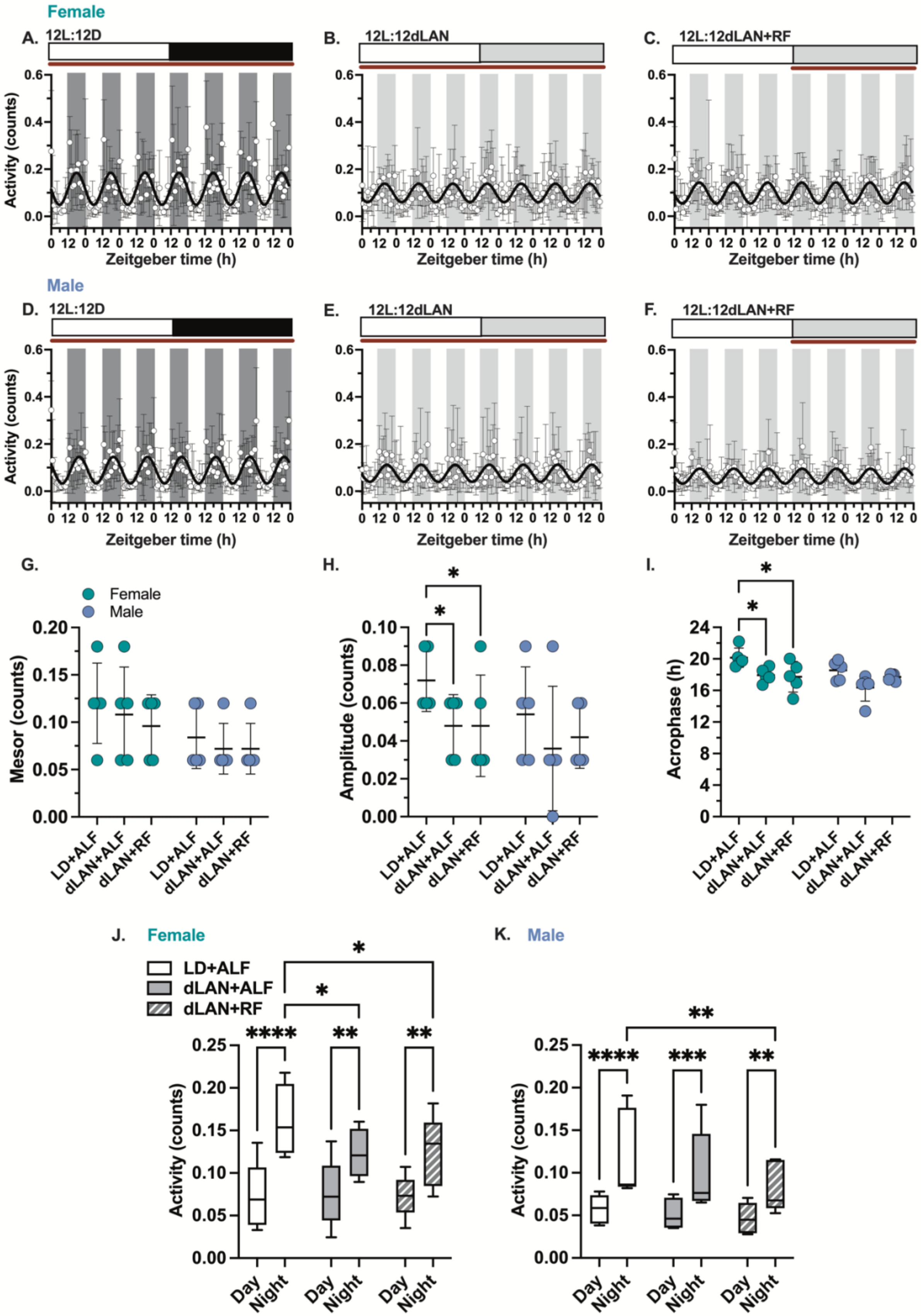

Female
A. 12L:12D
B. 12L:12dLAN
C. 12L:12dLAN+RF
Activity (counts)
Zeitgeber time (h)
Male
D. 12L:12D
E. 12L:12dLAN
F. 12L:12dLAN+RF
G.
Female
Male
Mesor (counts)
H.
Amplitude (counts)
I.
Acrophase (h)
LD+ALF
dLAN+ALF
dLAN+RF
J. Female
K. Male
LD+ALF
dLAN+ALF
dLAN+RF
Day
Night

Supplementary Figure 6: dLAN blunts amplitude of 24-hour activity rhythm

The hourly mean activity data recorded from female and male mice were plotted as a function of zeitgeber time for seven consecutive days. **A., D.** Show the data measured from female and male mice housed in LD+ALF (12-hour light: 12 hours dark; 200 lux: 0 lux; ad libitum feeding), **B., E.** show the data measured from female and male mice housed in dLAN+ALF (12-hour light: 12-hour dim light at night; 200 lux: 5 lux; ad libitum feeding) and **C., F.** show the data measured from female and male mice housed in dLAN+RF (12-hour light: 12 hours dim light at night; 200 lux: 5 lux; dim light restricted feeding) (n=5/sex). The shaded regions in the graphs correspond to the dark or dim light cycles. The inset above each graph shows the food accessibility (brown line) as a function of the LD or dLAN cycle. The individual female and male mouse time series data for the activity were fit to a cosine wave to calculate the amplitude (**G.**), mesor (**H.**), and acrophase (**I.**) for each condition. Data are presented as a scatter plot with the mean and SD. **J., K.** Show the average day and night activity in female and male mice (n=5/sex) under LD+ALF, dLAN+ALF, and dLAN+RF. Data are presented as box-whisker plots (median) with error bars representing minimum and maximum values. Significance was determined using a 2-way repeated measures ANOVA and Tukey's posthoc test. *$p<0.05$, **$p<0.01$, ***$p<0.001$, and ****$p<0.0001$.
