# Supplementary material for: Dim light at night unmasks sex-specific differences in circadian and autonomic regulation of cardiovascular physiology
Source: Commun Biol. 2024 Sep 27;7:1191. doi: 10.1038/s42003-024-06861-8 (PMC11437115; doi:10.1038/s42003-024-06861-8)
Supplement: Supplementary file 3 — Description of Additional Supplementary File [file 42003_2024_6861_MOESM3_ESM.pdf]

Description Of Additional Supplementary File

File name: Supplementary Data

Description: Detailed statistics of figures
