# Supplementary material for: Dim light at night unmasks sex-specific differences in circadian and autonomic regulation of cardiovascular physiology
Source: Commun Biol. 2024 Sep 27;7:1191. doi: 10.1038/s42003-024-06861-8 (PMC11437115; doi:10.1038/s42003-024-06861-8)
Supplement: Supplementary file 5 — Reporting Summary [file 42003_2024_6861_MOESM5_ESM.pdf]

nature portfolio

Corresponding author(s): DBPR COMMSBIO-24-3268B

Last updated by author(s): Aug 26, 2024

Reporting Summary

Nature Portfolio wishes to improve the reproducibility of the work that we publish. This form provides structure for consistency and transparency in reporting. For further information on Nature Portfolio policies, see our Editorial Policies and the Editorial Policy Checklist.

Statistics

For all statistical analyses, confirm that the following items are present in the figure legend, table legend, main text, or Methods section.

n/a	Confirmed	
☐	☒	The exact sample size (*n*) for each experimental group/condition, given as a discrete number and unit of measurement
☐	☒	A statement on whether measurements were taken from distinct samples or whether the same sample was measured repeatedly
☐	☒	The statistical test(s) used AND whether they are one- or two-sided<br>*Only common tests should be described solely by name; describe more complex techniques in the Methods section.*
☐	☒	A description of all covariates tested
☐	☒	A description of any assumptions or corrections, such as tests of normality and adjustment for multiple comparisons
☐	☒	A full description of the statistical parameters including central tendency (e.g. means) or other basic estimates (e.g. regression coefficient) AND variation (e.g. standard deviation) or associated estimates of uncertainty (e.g. confidence intervals)
☐	☒	For null hypothesis testing, the test statistic (e.g. *F*, *t*, *r*) with confidence intervals, effect sizes, degrees of freedom and *P* value noted<br>*Give P values as exact values whenever suitable.*
☒	☐	For Bayesian analysis, information on the choice of priors and Markov chain Monte Carlo settings
☒	☐	For hierarchical and complex designs, identification of the appropriate level for tests and full reporting of outcomes
☒	☐	Estimates of effect sizes (e.g. Cohen's *d*, Pearson's *r*), indicating how they were calculated

Our web collection on statistics for biologists contains articles on many of the points above.

Software and code

Policy information about availability of computer code

Data collection	Data were collected with Ponemah Softwar (Data Science International) and Clocklab Chamber Control Software (Actmetrics)
Data analysis	GraphPad PRISM Version 10.2.0<br>ClockLab Analysis Software

For manuscripts utilizing custom algorithms or software that are central to the research but not yet described in published literature, software must be made available to editors and reviewers. We strongly encourage code deposition in a community repository (e.g. GitHub). See the Nature Portfolio guidelines for submitting code & software for further information.

Data

Policy information about availability of data

All manuscripts must include a data availability statement. This statement should provide the following information, where applicable:

- Accession codes, unique identifiers, or web links for publicly available datasets
- A description of any restrictions on data availability
- For clinical datasets or third party data, please ensure that the statement adheres to our policy

The data that support the findings of this study are available from the corresponding author upon reasonable request.

Human research participants

Policy information about studies involving human research participants and Sex and Gender in Research.

Reporting on sex and gender	Not appplicable
Population characteristics	Not applicable
Recruitment	Not applicable
Ethics oversight	Not applicable

Note that full information on the approval of the study protocol must also be provided in the manuscript.

Field-specific reporting

Please select the one below that is the best fit for your research. If you are not sure, read the appropriate sections before making your selection.

☒ Life sciences ☐ Behavioural & social sciences ☐ Ecological, evolutionary & environmental sciences

For a reference copy of the document with all sections, see nature.com/documents/nr-reporting-summary-flat.pdf

Life sciences study design

All studies must disclose on these points even when the disclosure is negative.

Sample size	We found Sample size of n=6 per group per sex was optimal for our studies since ECG and Blood pressure studies uses repeated measures analysis based on time and conditions.
Data exclusions	We excluded one ECG and Blood pressure parameters of each sex due to irregularity in the data.
Replication	We repeated our experiment twice (one as a pilot experiment) to generate preliminary data and found similar results in further experiments.
Randomization	The randomization was not relevant to this study. It was done on the wild-type male and female mice of similar age group.
Blinding	The analysis of feeding, ECG and blood pressure were done by researchers blind to the experimental conditions and protocol.

Reporting for specific materials, systems and methods

We require information from authors about some types of materials, experimental systems and methods used in many studies. Here, indicate whether each material, system or method listed is relevant to your study. If you are not sure if a list item applies to your research, read the appropriate section before selecting a response.

Materials & experimental systems

n/a	Involved in the study	
☒	☐	Antibodies
☒	☐	Eukaryotic cell lines
☒	☐	Palaeontology and archaeology
☐	☒	Animals and other organisms
☒	☐	Clinical data
☒	☐	Dual use research of concern

Methods

n/a	Involved in the study	
☒	☐	ChIP-seq
☒	☐	Flow cytometry
☒	☐	MRI-based neuroimaging

Animals and other research organisms

Policy information about studies involving animals; ARRIVE guidelines recommended for reporting animal research, and Sex and Gender in Research

Laboratory animals	SV129 Mice (Mus musculus); Male and female-4-6 months old
Wild animals	This study did not involve wild animals.

Reporting on sex	Sex is used as a biological variable throughout the study.
Field-collected samples	This study did not involve field collected samples.
Ethics oversight	All animal procedures complied with the Association for Assessment and Accreditation of Laboratory Animal Care guidelines with approval by the Institutional Animal Care and Use Committee (2019-3304) at the University of Kentucky.

Note that full information on the approval of the study protocol must also be provided in the manuscript.
